# Effects of Simulated Nitrogen and Phosphorus Deposition on Dioecious *Populus cathayana* Growth and Defense Traits

**DOI:** 10.3390/plants14081261

**Published:** 2025-04-21

**Authors:** Junyu Li, Yongmei Liao, Wanrong Wei, Xiaoqin Xu, Jundong He, Tingting Zhao

**Affiliations:** 1 College of Geography and Geomatics, Xuchang University, Xuchang 461000, China; lijunyu0506@126.com; 2 Key Laboratory of Southwest China Wildlife Resources Conservation, Ministry of Education, Nanchong 637009, China; lym3326@126.com (Y.L.); weiwr18@126.com (W.W.); xuxiaoqin21@126.com (X.X.); zhaotingt2019@163.com (T.Z.); 3 College of Pastoral Agriculture Science and Technology, Lanzhou University, Lanzhou 730000, China; 4 Institute of Ecology, China West Normal University, Nanchong 637009, China

**Keywords:** N and P deposition, dioecy, defense substances, growth–defense traits

## Abstract

Human activities have increased the imbalance in atmospheric N and P deposition, which changes soil nutrient availability and subsequently affects the structure and function of terrestrial ecosystems. Dioecious plants are important parts of terrestrial ecosystems and are characterized by sex-related differences in their response to the external environment and always exhibit a skewed sex ratio, which makes them more vulnerable to climate change and increases their risk of extinction. However, little attention has been paid to the effects of unbalanced N and P deposition on these plants, especially on their defense traits. In this study, we used dioecious *Populus cathayana* to investigate the influence of gradient N and P deposition on the correlation between growth and defense traits. The results showed that although the different rates of N and P deposition enhanced biomass accumulation in both sexes to varying degrees, the most substantial biomass increment was noted under a lower-nitrogen and higher-phosphorus (LNHP) treatment regimen, with females showing an approximately 112% increase and males a 47% increase in total biomass. In response to varying levels of simulated N and P deposition, males and females adopt distinct strategies for biomass allocation. Although declines in root biomass were observed in both sexes as nutrient availability increased, the decrement was more marked in males; under the LNHP treatment, it dropped by about 11%, while under a high-nitrogen and high-phosphorus (HNHP) treatment, the decrease was about 35%. Conversely, females demonstrated a heightened propensity to allocate biomass towards leaf development. Furthermore, with increasing N and P deposition, there was a general reduction in the concentrations of physical and chemical defense substances within the leaves of both sexes. Nonetheless, the correlations between defense substances, nutrient element content, non-structural carbohydrate (NSC) content, and dry biomass were more pronounced in males, suggesting a greater sensitivity to defense substance responses in males than in females. Overall, these results indicate that there is sexual dimorphism in the accumulation of chemical substances in male and female *P. cathayana* under unbalanced N and P deposition and they provide a technical and theoretical basis for predicting the population dynamics of dioecious plants, maintaining the stability of poplar populations, and constructing high-productivity poplar plantations globally in the future.

## 1. Introduction

Dioecious plants, which constitute approximately 6% of all plant species [1], are integral to the dynamics of ecosystems. They optimize resource use and reduce intraspecific competition via niche differentiation between sexes, thereby enhancing population adaptability [2]. Their underground ecological interactions, such as sex-specific mycorrhizal associations, improve soil health and stability [3]. Additionally, these plants also show sex-specific stress resistance, which allows them to survive in diverse environments by creating more ecological niches and bolstering ecosystem stability [4]. Therefore, they are essential for preserving species diversity within terrestrial ecosystems and for upholding the stability of forest ecosystem functions [5,6]. Studies have shown that dioecious plants commonly exhibit sexual dimorphism in terms of their phenology, morphology, physiology, and defense mechanisms, showcasing sex-specific responsiveness to environmental changes, but not in their reproductive organs [2,7]. As a result, these plant populations often deviate from the theoretical 1:1 sex ratio [1], making them more vulnerable to the effects of environmental variability and potentially heightening the risk of population extinction [8].

Anthropogenic activities have led to the atmospheric deposition of nitrogen (N) and phosphorus (P), with the imbalance in atmospheric N and P deposition being a particular point of interest [9]. As the primary nutrient factors limiting plant growth in terrestrial ecosystems, N and P play a crucial role in the growth, development, and reproduction of plants. The balance between N and P is also essential for maintaining the stability of terrestrial ecosystems [10,11]. Studies have shown that although a certain degree of N and P deposition can enhance the availability of soil N and P elements, alleviate the nutrient limitation of ecosystems, and promote plant growth [12], a continuous imbalanced deposition of N and P can lead to numerous negative effects. These include soil acidification [13], the imbalance of soil N and P exacerbating the P limitation on plant growth [14,15,16], and impacting plant growth and defense mechanisms, which in turn exacerbates plant diseases and pests, etc. [17].

Existing studies indicate that female and male *Populus* trees employ distinct adaptation strategies in response to variations in N and P supply, with females exhibiting increased sensitivity to enhanced N and P availability and males showing a higher tolerance for N and P deficiencies. Under N and P deficiency conditions, although the photosynthetic rate of both female and male *Populus* trees decreases significantly, male trees maintain a higher photosynthetic rate, nutrient use efficiency, and stronger resistance to nutrient stress [18]. Moreover, under P deficiency, male trees secrete more acidic phosphatase and have a higher arbuscular mycorrhizal fungal biomass, endowing them with a stronger P acquisition capacity [19]. Metabolic and physiological research also reveals that male trees adopt energy-saving strategies to cope with P deficiency environments [20]. Consequently, under N and P deficiency conditions, the sex ratio of *Populus* populations skews towards males, which is consistent with the findings of existing research on dioecious plants [21].

In contrast, under simulated N deposition (simulated levels of 0, 75, and 150 kg ha^−1^ a^−1^), female and male *P. cathayana* exhibited significant sex-specific responses. Females demonstrated stronger growth and enhanced photosynthetic efficiency in the nitrogen deposition environment. Especially at high nitrogen levels, their plant height, basal diameter, total leaf area, and dry matter accumulation significantly increased. Meanwhile, their photosynthetic nitrogen use efficiency (PNUE) was also higher, which indicated that females were more adept at acquiring and utilizing resources in nitrogen-rich environments [22]. On the other hand, under conditions of increased P availability, the total root length, specific root length, biomass, and leaf P content of female trees all increased significantly, and this increase was also significantly greater than that in male trees [19]. In addition, under N deposition, due to their higher light capture capacity (larger leaf area) and photosynthetic N use efficiency (PNUE), female *Populus* trees gained an advantage in interspecific competition [22,23]. However, the changes in the sex bias of female and male *Populus* populations under unbalanced atmospheric N and P deposition remain unclear.

Furthermore, before reaching sexual maturity, male and female plants exhibit differences in physiological, morphological, and defensive mechanisms, indicating that these differences are congenital and not solely caused by the costs of reproduction after sexual maturity [19]. According to the previous study, *P. cathayana* consistently maintains a tradeoff between growth and defense from the juvenile stage to maturity, and this tradeoff is closely related to the sex of the plant [6]. In addition, males and females show plasticity in the defensive compounds metabolism, which could be adjusted according to external environmental variation [7]. For example, warming decreased herbivore-induced responses in defensive compounds, such as salicortin, condensed tannins, salicylic acid, jasmonic acid and defensive-related enzyme activities more in females than in males, which finally increased consumed leaf area in females more in males [6]. Another study on the dioecious *P. tremula* has revealed that the content and stoichiometric relationship of N and P elements could influence plant defense functions through secondary metabolic processes. The results revealed that N had more influence on growth and phenolic concentrations of *P. tremula* seedlings than P, while P had more influence on the accumulation of leaf flavonoid-derived phenylpropanoids than N, in addition, P limitation has a greater impact on leaf defense substances than nitrogen limitation, with a more significant effect observed in female plants [24]. However, there is relatively little research on how an increasingly intensified and imbalanced deposition of N and P affects plant growth and defense functions, and studies specifically targeting dioecious plants are even rarer.

*Salicaceae* plants are common dioecious plants, widely used in ecological management, environmental improvement, and wood processing because of their strong adaptability, easy reproduction, and rapid growth. Therefore, *Salicaceae* plants are also important fast-growing afforestation trees worldwide. Studies on the adaptation mechanisms of male and female plants to global climate change and environmental stress—and on the relevant sex differences—using *Populus* as a model tree species have found that female plants are more sensitive to a stressed environment, while male plants are more resilient. Therefore, the sex ratio of the natural population of poplar trees is usually male-skewed [25]. Of note, there are relatively few studies on the adaptation mechanisms of dioecious poplar and even fewer studies on its growth–defense function tradeoff.

Against the background of increasing N and P deposition imbalance, it is of great value to explore the physiological and biochemical response mechanisms of dioecious plants and the tradeoff between their growth and defense function for predicting and maintaining the dynamics and stability of dioecious plant populations during global changes. The differences in the strategies of physiological and ecological responses to environmental changes at the individual level in dioecious plants provide an ideal research model for exploring the allocation of plant defense resources [24]. In the current study, with *P. cathayana* used as the research object, we aim to the answer the following questions: (1) How do unbalanced N and P supply levels affect the growth–defense traits of dioecious *P. cathayana*? (2) What is the internal mechanism of adaptation to unbalanced N and P deposition? (3) Is there a sex-specific adaptation to unbalanced N and P deposition?

## 2. Results

### 2.1. Biomass Accumulation and Leaf Areas

**Figure 1:**
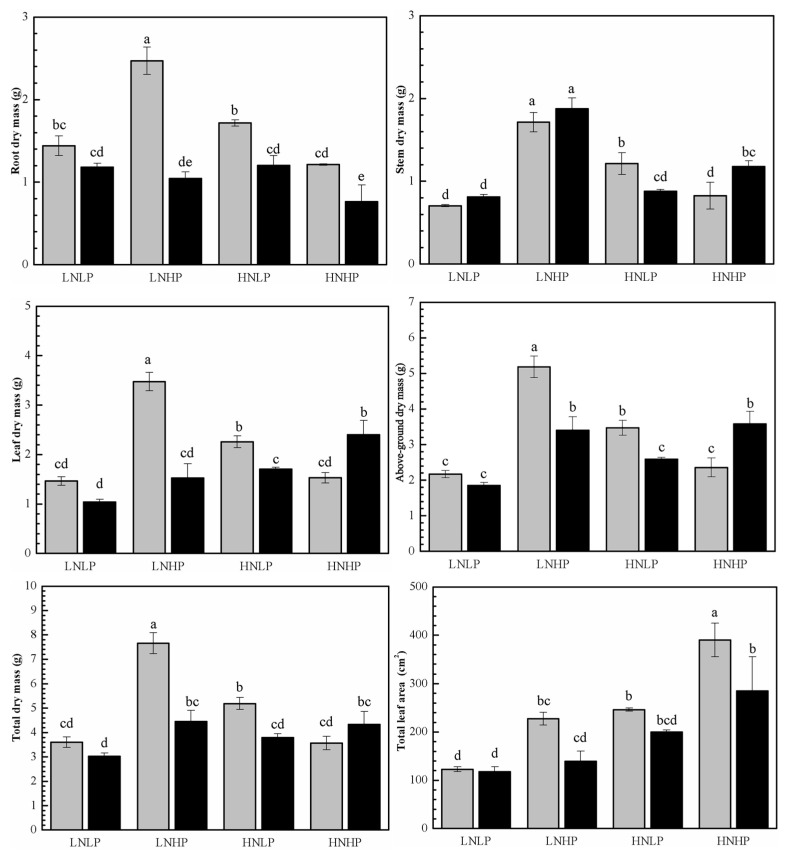
The biomass accumulation of different organs and total leaf area in female (gray) and male (black) *P. cathayana* seedlings as affected by different N and P supply. Each value is the mean ± SE (n = 3). The values not sharing the same letters are significantly different at *p* < 0.05 according to Duncan’s tests for one-way ANOVAs. LNLP, low N (0.4 mM) and low P (0.002 mM) concentration composition; LNHP, low N (0.4 mM) and high P (1 mM) concentration composition; HNLP, high N (5 mM) and low P (0.002 mM) concentration composition; and HNHP, high N (5 mM) and high P (1 mM) concentration composition.

Under a lower-nitrogen and lower-phosphorus (LNLP) treatment, no significant sex-based differences in biomass accumulation or leaf area were observed. However, under an LNHP treatment, both sexes exhibited a substantial increase in biomass accumulation across all organs and in total biomass. Notably, females showed significantly higher above-ground, leaf, and total biomass accumulation compared to males. Conversely, under a higher-nitrogen and lower-phosphorus (HNLP) treatment, females demonstrated a remarkable enhancement in biomass accumulation and leaf area, while males only showed a significant increase in leaf biomass accumulation. Significantly, under the HNLP treatment, females accumulated markedly more biomass than males. Furthermore, the HNHP treatment resulted in a significant reduction for males, whereas for females, this treatment had no significant impact on these parameters. Under the HNHP treatment, significant sexual differences were observed in stem, leaf, above-ground, and root biomass. Additionally, females reached peak total biomass accumulation under the LNHP treatment, whereas no significant differences were detected among the HNLP, HNHP, and LNLP treatments for males (Figure 1, Table 1).

### 2.2. Biomass Distribution

**Figure 2:**
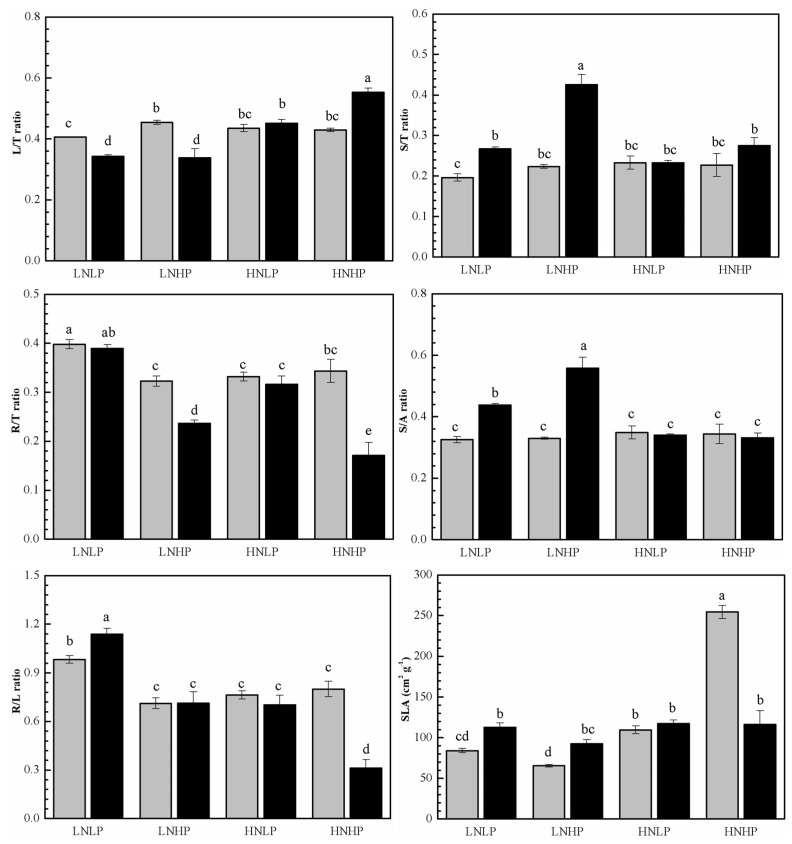
The biomass distribution of different organs and SLA in female (gray) and male (black) *P. cathayana* seedlings as affected by different N and P supply. Each value is the mean ± SE (n = 3). The values not sharing the same letters are significantly different at *p* < 0.05 according to Duncan’s tests for one-way ANOVAs. LNLP, low N (0.4 mM) and low P (0.002 mM) concentration composition; LNHP, low N (0.4 mM) and high P (1 mM) concentration composition; HNLP, high N (5 mM) and low P (0.002 mM) concentration composition; and HNHP, high N (5 mM) and high P (1 mM) concentration composition.

Under the LNLP treatment, significant sexual differences in biomass allocation were observed. Specifically, females exhibited a higher L/T ratio compared to males, while the S/T, R/T, and R/L ratios and SLA were significantly lower in females than in males. Compared with the LNLP treatment, the LNHP treatment significantly increased the L/T ratio in females and the S/T and R/T ratios in males while decreasing the R/L ratio of both sexes. Under the LNHP treatment, the R/T and L/T ratios were significantly higher in females than in males, but the S/T ratio, S/A ratio, and SLA were significantly lower. Compared with the LNLP treatment, the HNLP treatment significantly increased the L/T ratio in males and the SLA in females while reducing the S/A ratio in males and the R/T and R/L ratios in both sexes. No significant sex differences in biomass allocation indices were observed under the HNLP treatment. Again, compared with the LNLP treatment, the HNHP treatment significantly increased the L/T ratio in males and the SLA in females, while it decreased the S/A ratio in males and the R/T ratio in females. Under the HNHP treatment, the L/T ratio in males was lower but the R/T ratio, R/L ratio, and SLA were significantly higher in males than in females (Figure 2, Table 1).

### 2.3. C, N, and P Content and the Ratios

**Figure 3:**
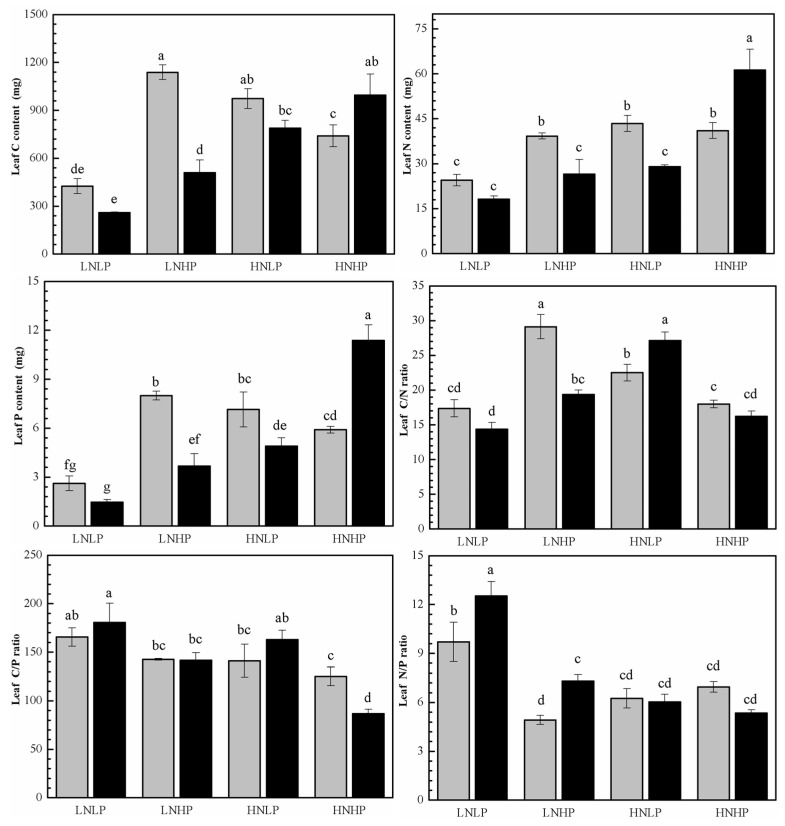
Foliar C, N, and P content in female (gray) and male (black) *P. cathayana* seedlings as affected by different N and P supply. Each value is the mean ± SE (n = 3). The values not sharing the same letters are significantly different at *p* < 0.05 according to Duncan’s tests for one-way ANOVAs. LNLP, low N (0.4 mM) and low P (0.002 mM) concentration composition; LNHP, low N (0.4 mM) and high P (1 mM) concentration composition; HNLP, high N (5 mM) and low P (0.002 mM) concentration composition; and HNHP, high N (5 mM) and high P (1 mM) concentration composition.

Under the LNLP treatment, there were no significant sex differences in C, N, and P contents or ratios in the leaves of plants of both sexes, except that the N/P ratio was significantly lower in females than in males. Compared with LNLP treatment, both the LNHP and HNLP treatments substantially increased the C content, P content, and C/N ratio in the leaves of plants of both sexes; they also significantly increased the N content but decreased the N/P ratio in female leaves. Specifically, under the LNHP treatment, the C, N, and P contents and the C/N ratio in female leaves were consistently higher than those of males, whereas the N/P ratio was markedly lower than that of males. Conversely, under the HNLP treatment, the N and P contents in females were higher than those in males, but the C/N ratio was significantly lower. Furthermore, compared with the LNLP treatment, the HNHP treatment increased the C, N, and P contents in the leaves of female plants, while it significantly reduced their C/P and N/P ratios. Additionally, under the HNHP treatment, C, N, and P contents in females were lower than those in males, but the C/P ratio was significantly higher (Figure 3, Table 1).

### 2.4. Carbohydrate Content

**Figure 4:**
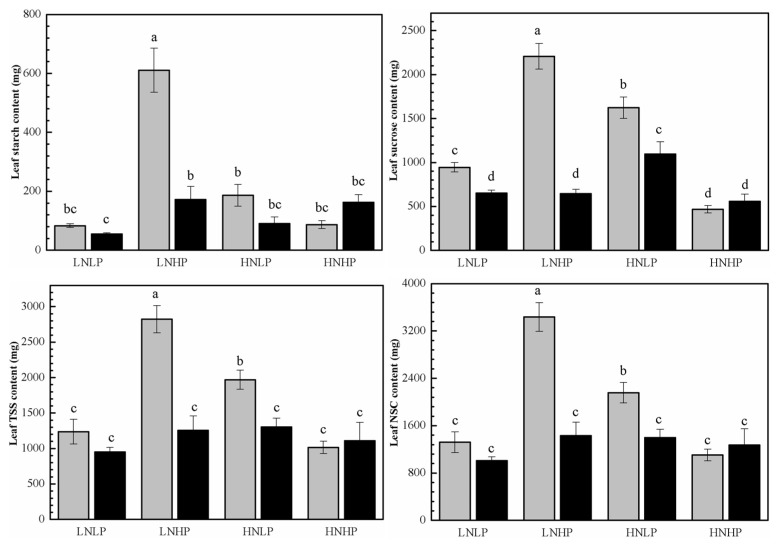
Foliar starch, sucrose, total soluble sugar (TSS) and non-structural carbohydrates (NSC) content in female (gray) and male (black) *P. cathayana* seedlings as affected by different N and P supply. Each value is the mean ± SE (n = 3). The values not sharing the same letters are significantly different at *p* < 0.05 according to Duncan’s tests for one-way ANOVAs. LNLP, low N (0.4 mM) and low P (0.002 mM) concentration composition; LNHP, low N (0.4 mM) and high P (1 mM) concentration composition; HNLP, high N (5 mM) and low P (0.002 mM) concentration composition; and HNHP, high N (5 mM) and high P (1 mM) concentration composition.

Under the LNLP treatment, there were no significant sexual differences in carbohydrate content in the leaves of plants of both sexes, except for in female leaves, where the sucrose content was significantly higher than in males. Compared with the LNLP treatment, both the LNHP and HNLP treatments substantially increased the TSS, sucrose, starch, and NSC contents in female leaves, with the LNHP treatment boosting the starch content and the HNLP treatment enhancing the sucrose content in male leaves. Moreover, under both treatments, females exhibited significantly higher carbohydrate contents compared to males. Conversely, the HNHP treatment exhibited no significant effect on carbohydrate content in either sex, except for the sucrose content in females, which was significantly decreased (Figure 4, Table 1).

### 2.5. Content of Defensive Substances and APA Activity in Leaves

**Figure 5:**
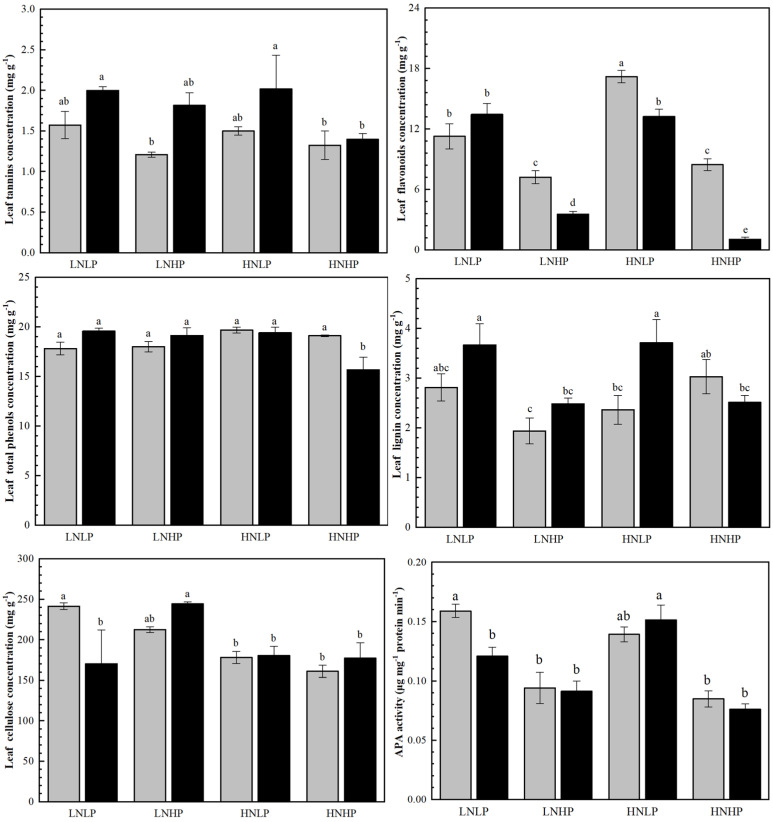
Foliar defensive substance content in female (gray) and male (black) *P. cathayana* seedlings as affected by different N and P supply. Each value is the mean ± SE (n = 3). The values not sharing the same letters are significantly different at *p* < 0.05 according to Duncan’s tests for one-way ANOVAs. LNLP, low N (0.4 mM) and low P (0.002 mM) concentration composition; LNHP, low N (0.4 mM) and high P (1 mM) concentration composition; HNLP, high N (5 mM) and low P (0.002 mM) concentration composition; and HNHP, high N (5 mM) and high P (1 mM) concentration composition.

Under the LNLP treatment, the cellulose content and APA activity in female leaves were significantly higher than those in males, while no significant sex differences were observed in other defense compounds. Compared to LNLP, the LNHP treatment decreased the flavonoid content in plants of both sexes and the lignin content in males while increasing cellulose content in females; the HNLP treatment increased the flavonoid content in females. In contrast, the HNHP treatment significantly decreased the contents of all defense compounds in males, as well as cellulose content in females. Overall, N treatments had almost no significant effect on defense compounds in dioecious *Populus*, whereas P treatments showed the opposite trend. Additionally, in the latter three nutrient-enhanced treatments, the flavonoid content in females was consistently higher than that in males (Figure 5, Table 1).

### 2.6. Pearson Correlation Coefficients Between Leaf Defensive Parameters and Nutrient and Carbohydrate Contents

For male plants, the contents of tannins, flavonoids, and total phenolic substances in leaves were significantly negatively correlated with N and P content. The total phenolic and flavonoid contents were significantly negatively correlated with leaf dry weight, while flavonoids were significantly positively correlated with the contents of soluble sugars, sucrose, and NSCs. Lignin content was significantly negatively correlated with starch content, whereas cellulose and starch contents were significantly positively correlated. In contrast, for female plants, lignin content in leaves was significantly negatively correlated with C content, leaf dry weight, and NSC content; cellulose content was significantly negatively correlated with N content. Additionally, in male plants, flavonoid content was significantly positively correlated with lignin, total phenolic, and tannin contents, while in female plants, total phenolic content was significantly positively correlated with cellulose content (Table 2).

## 3. Discussion

In recent years, China has become one of the three regions with the highest N deposition rates in the world [26]. The total N deposition level increased from 13.2 kg hm^−2^ a^−1^ in the 1980s to 21.1 kg hm^−2^ a^−1^ in the 2000s [27]. The maximum and average values of total N deposition in North China are 106.5 kg hm^−2^ a^−1^ and 54.5 ± 17.2 kg hm^−2^ a^−1^, respectively [28]. However, the N and P deposition rates in China are unbalanced [29]: the average atmospheric N and P deposition values are 13.69 ± 8.69 kg N ha^−1^ a^−1^ and 0.21 ± 0.17 kg P ha^−1^ a^−1^, respectively, and the average N/P ratio is 77. Based on the fundamental principles of stoichiometry, this might enhance the limiting effect of phosphorus on ecosystems [30]. It has also been verified that the balance between nutrient elements is even more important than their absolute content; this is especially the case for N and P, elements necessary for plant growth [31]. Numerous simulated N and P addition experiments have been carried out worldwide to explore the effects of imbalanced N and P deposition on the dynamics of N and P in the soil and in plants [15,32]. Given the current severely unbalanced N and P sedimentation, it is imperative to investigate the response mechanisms and sex-specific attributes of dioecious plants.

### 3.1. The Effects on Biomass Accumulation

In our investigation, compared with lower N and P deposition (LNLP treatment), individual high N deposition (HNLP) and high P deposition (LNHP) and simultaneous high N and P deposition (HNHP) all increased biomass accumulation and leaf area in both sexes to varying degrees, with the exception of root dry weight (Figure 1). These results are consistent with previous studies [33,34]. Since it is generally recognized that terrestrial ecosystems are constrained by the availability of N and P, additional N and P deposition could promote plant growth by alleviating N limitation and increasing biomass accumulation [10,35]. However, in female plants, the most prominent biomass accumulation occurred under the LNHP treatment, followed by the HNLP treatment. In contrast, although the HNLP treatment exhibited a trend of biomass increase, its growth margin did not reach a statistically significant level compared to the LNLP treatment. The existing literature indicates that, when N deposition is excessive, although the N content in plant leaves also increases as a result, the additional and redundant N is not used to enhance photosynthetic capacity. Instead, it accumulates in the plant in the form of putrescine, a stress indicator, or its precursor arginine [36]. In conclusion, the biomass accumulation results suggest that sustained imbalanced N and P deposition might lead to adverse effects on plant growth. Such a conclusion has already been validated in previous studies [15,16,17].

### 3.2. The Effects on Biomass Distribution

According to the optimal allocation theory, plants tend to allocate a large proportion of biomass in the organs, which limits their growth [34]. This suggests that with an in-crease in external N availability, plants will tend to reduce their nutrient investment in below-ground components to enhance their capacity for utilizing photosynthetic re-sources, thereby resulting in a lower root-to-shoot ratio [37,38]. In our experiment, both sexes exhibited similar changes in biomass distribution alteration from below- to above-ground organs. These results are consistent with previous studies [33]. Nevertheless, under different simulated rates of N and P deposition, the two sexes exhibited radically different above-ground biomass distribution patterns between the leaves and the stem. The findings indicate that under higher nutrient deposition, males had a tendency towards stem biomass distribution, while females had a tendency towards leaf biomass distribution. Additionally, male roots were more sensitive to variation in N and P availability under simulated deposition, and the phenomenon of a reduced allocation of root biomass was particularly prominent (Figure 2). In addition, the significant root biomass distribution in males indicated a greater flexibility of root plasticity, especially under P deposition. Unlike the males, although female plants tended to distribute more biomass to the leaves under simulated nutrient depositions, the results for SLA varied: under the LNHP treatment, despite a significant increment in leaf biomass allocation and leaf area, SLA significantly decreased, whereas under the HNLP and HNHP treatments, SLA was significantly increased, which indicated that the leaf area of female plants is more sensitive to the availability of N and that females tend to maximize their photosynthetic area to ensure their growth requirements are met [19,22,23]. Considering the different physiological functions of different organs, these distinctive biomass distribution patterns between the two sexes also reflect their varied adaptive mechanisms to N and P deposition.

Studies on the Resource Availability Hypothesis (RAH) suggest that in resource-poor environments, plants tend to increase the synthesis of their defensive substances, correspondingly slowing down their growth rate, while in resource-abundant conditions, plants will reduce their investment in defensive mechanisms, thus promoting their growth [39]. However, there is also research showing that in resource-rich environments, plants have the capacity to simultaneously increase their growth rate and their investment in defensive resources [40], and some studies even indicate that there is a positive correlation between plant growth and defensive functions [41]. Leaves are considered to be the most sensitive organs when it comes to the availability of plant nutrients [42]. In the present study, we investigated the response of foliar nutrient contents, NSC contents, and defensive substance contents to simulated N and P depositions. The results showed that the simulated N and P deposition treatments resulted in significant increments in leaf C, N, P, and NSC contents to different extents. These increases were consistent with the biomass accumulation patterns in both sexes; on the other hand, these results were not entirely consistent with the variation in defensive substances in the leaves of both sexes.

### 3.3. The Effects on Leaf Defensive Traits

Evidence shows that N and P could be involved in regulating plant secondary metabolic processes and affect plant defensive functions [24]. For dioecious plants, due to the higher reproductive investment of female plants, the resource allocation for growth and defense is bound to decrease. Thus, it is crucial for female plants to enhance their defense capabilities to protect their growth potential and prevent tissue loss [43]. Female plants have stronger defense abilities against animals, which has been verified in multiple species [44]. In the present study, the contents of the majority of defense substances decreased. Combining the growth-related indicators and the variation in defense substances, the findings of this research study are consistent with the predictions of the RAH [39]. However, there were sex-specific differences among defensive substances in *P. cathayana* under simulated N and P depositions (Figure 5). According to the Pearson correlation analysis, in males, the relative parameters were more correlated to their physiological parameters, and the chemical and physical defensive substances were more sensitive to N and P availability compared to females. On the contrary, in females, physical defensive substances were more stable than chemical defensive substances, regardless of N and P availability; lignin and cellulose concentrations showed no obvious variation under the simulated nutrient depositions, except for cellulose under the HNHP treatment (Figure 5). The findings were largely in line with the results of previous research holding that females and males had differences in leaf metabolic profile where females typically allocate more resources to chemical defense and tolerance to insect herbivory feeding when compared with males [7,25]. Compared with chemical defenses, physical defenses seem to be less costly—in the long run—and more effective against herbivory [45]. Thus, under sustained N and P deposition, female *P. cathayana* might benefit from the stability of its physical defenses.

## 4. Materials and Methods

### 4.1. The Plant Material and Hydroponic Experimental Design

**Figure 6:**
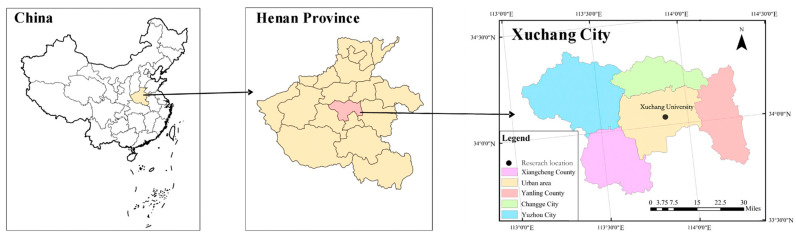
The location map of where the research was conducted.

Healthy annual cuttings of *P. cathayana* Rehd. were collected from different male and female stock plants in riparian and flat valley habitats located in Qinghai, China (Datong, 35°560′ N, 101°350′ E). To ensure the randomness and independence of experimental treatments, each cutting was obtained from a different individual parent tree. The male and female cuttings were planted separately in the field, under ambient conditions, at Xuchang University campus in March 2019 in Xuchang city (34°16′–34°58′ N, 112°42′–114°14′ E), which is located in the central part of Henan Province (China) and has a temperate continental monsoon climate (Figure 6). The daytime average temperature during the treatment period was 10–21 °C, the night-time average temperature was 8–16 °C, the average annual precipitation from 671 to 736 mm, and the relative humidity was 40–85%. After sprouting and growing for about two months, 40 male and 40 female healthy cuttings with a similar crown size (~15 leaves) and uniform height (~30 cm) were chosen. The root systems were cleaned thoroughly, after which the cuttings were replanted in 10 L plastic pots (one cutting per pot) filled with liquid medium, which was a modified Hoagland solution. The nutrient solution composition was supplied according to the requirements of the experimental design, as described below. Except the N and P elements, the types and concentrations of other elements followed those specified by Li et al. [46].

This experiment utilized a completely randomized design with eight factorial combinations of two levels each for sex (male and female), N (lower N and higher N), and P (lower P and higher P). The addition of N was primarily determined based on the atmospheric N deposition rates in North China [28]. Similarly, the addition of P was set in accordance with the P deposition rates cited in previous research [11,47]. NH_4_NO_3_ was used as the nitrogen source, while KH_2_PO_4_ served as the phosphorus source. Forty plots of each sex were divided into two lots and set in two N regimes. The first lot was fed with a nutrient solution with the addition of 0.4 mM NH_4_NO_3_ (LN), and the second lot was fed with a nutrient solution with the addition of 5 mM NH_4_NO_3_ (HN). Then, the cuttings from each treatment were divided into two batches and subjected to two P treatments. The first lot was fed with a nutrient solution with the addition of 0.002 mM KH_2_PO_4_ (LP), and the second lot was fed with a nutrient solution with the addition of 1 mM KH_2_PO_4_ (LP). Thus, each sex was subjected to four treatments: (1) lower N and lower P (LNLP); (2) lower N and higher P (LNHP); (3) higher N and lower P (HNLP); and (4) higher N and higher P (HNHP). Each treatment included ten cuttings. In the eight different treatments, each cutting was grown in a separate plastic pot containing 10 L of the corresponding nutrient solution. The nutrient solution was changed every 3 days. The details of the composition of the nutrient solution in terms of the different nitrogen and phosphorus combinations are shown in Table 3. The main nutrient contents, such as sulfur, phosphorus, potassium calcium, and magnesium, were the same among all treatments. The pH of the nutrient solutions was adjusted to 6.0 with HCl or NaOH. Pots were shifted within treatments every week to eliminate location effects. The treatments started on 20 May 2019, and plants were harvested on 20 August 2019. At the end of the experiment, the fourth and fifth fully expanded leaves (counted from the top of each plant) were collected to be used for the analyses.

### 4.2. Growth Measurements

At the end of the experiment, three cuttings were randomly harvested from each treatment and partitioned into leaves, stems, and roots. All samples were dried separately at 80 °C to constant weight and weighed. The dried leaves (fourth to sixth leaves) were ground and used for element analysis. Leaf area (LA) was determined with a portable laser area meter (CI-203, CID Inc., Camas, WA, USA). The dry masses of the leaves (LM), stems (SM), and roots (RM) were measured. The total dry mass (TM), above-ground dry mass (AM), the ratios of LM/TM (L/T), SM/TM (S/T), SM/AM (S/A), RM/TM (R/T), and RM/LM (R/L), and specific leaf area (SLA, LA/LM) were also calculated.

### 4.3. Foliar C, N, and P Content Measurements

Dried samples were ground into a fine powder and passed through a mesh (pore diameter ca. 275 lm). C, N, and P concentrations were determined using the rapid dichromate oxidation technique [49], the semi-micro Kjeldahl method [50], and induced plasma emission spectroscopy [51], respectively. The C, N, and P content of each treatment was determined using the dry mass of each plant compartment. The total C-to-N ratio (C/N) and C-to-P ratio (C/P) were calculated as an estimate of long-term N and P use efficiency [52].

### 4.4. Foliar Total Soluble Sugar (TSS), Sucrose, Starch, and Non-Structural Carbohydrate Content Measurements

For the measurement of total soluble sugars, sucrose was extracted from dried leaves in 80% (*v/v*) ethanol. Total soluble sugars were detected colorimetrically at 625 nm following the anthrone–sulfuric acid method [53], while sucrose was detected colorimetrically at 480 nm following the modified resorcinol method [54]. The starch content in the pellet of plant material that remained was determined after the removal of ethanol [55]. Solutions were filtered through Whatman GF/C filters and diluted in 10 mL volumetric flasks. The concentrations of starch as glucose equivalents were determined colorimetrically. The absorption of an enzyme blank was subtracted from each sample’s absorbance prior to the calculation of the sugar content. The dry mass and concentrations of total soluble sugars, sucrose, and starch in leaves were used to calculate their contents, as well as the NSC content [56]. Acid phosphatase activity was assayed according to a previous method with some modifications, and absorbance was read at 405 nm using a spectrophotometer [57].

### 4.5. Foliar Defensive Substance Concentration Measurements: Tannins, Flavonoids, Total Phenols, Lignin, and Cellulose

The spectrophotometric method was used to determine the concentration of tannins. The absorbance was measured at a wavelength of 640 nm, and a standard curve was prepared using a (+)-Catechin hydrate + catechin standard solution with a concentration of 1.099 mg mL^−1^ [58]. The absorbance value of total flavonoids was measured at a 415 nm wavelength using a UV–Vis spectrophotometer [59]. The determination of total phenol concentration was conducted using spectrophotometry; absorbance was measured at a wavelength of 760 nm. A standard curve was prepared using a 1.000 mg/mL solution of tannic acid [60]. Finally, lignin and cellulose concentrations were measured gravimetrically using acetone and sulfuric acid [61].

## 5. Conclusions

Our study provides valuable insights into the sex-specific responses of dioecious *P. cathayana* to unbalanced N and P deposition, highlighting the significant impact of these environmental changes on the growth and defense traits of male and female plants. The results demonstrate that both sexes of *P. cathayana* exhibit distinct strategies for biomass allocation and defense mechanisms under varying N and P deposition conditions. Interestingly, female plants are more responsive to nutrient enrichment, particularly under conditions of higher phosphorus availability, and allocate more biomass towards leaf development, indicating a strategy focused on maximizing photosynthetic capacity and resource acquisition. In terms of defensive traits, our research found that the correlations between defense substances, nutrient element content, non-structural carbohydrate (NSC) content, and dry biomass were more pronounced in males, suggesting that males are more sensitive to changes in defense substance responses compared to females. This sexual dimorphism in the accumulation of chemical substances under unbalanced N and P deposition rates underscores the complexity of how dioecious plants adapt to changing environments. Despite the comprehensive insights provided by our study, several limitations should be acknowledged. First, the experiments were conducted under controlled conditions which may not fully replicate the complex and variable conditions found in natural ecosystems. Future studies should consider conducting field experiments to validate our findings under real-world conditions. Second, while we focused on the immediate responses of *P. cathayana* to N and P deposition, long-term studies are needed to understand the potential evolutionary and ecological consequences of these responses over multiple generations. Third, this study did not explore the underlying genetic and molecular mechanisms driving the observed sex-specific responses. Future research should integrate genomic and transcriptomic analyses to thoroughly elucidate the genetic basis underlying these adaptive strategies, providing deeper insights into their evolutionary mechanisms.

In conclusion, our findings highlight the importance of considering sex-specific responses when predicting the population dynamics of dioecious plants under global changes. These results provide a technical and theoretical basis for maintaining the stability of poplar populations and constructing high-productivity poplar plantations in the future. Subsequent research should focus on exploring the long-term ecological impacts and the genetic mechanisms underlying these responses to better understand and manage dioecious plant populations in changing environments.

## Figures and Tables

**Table 1:** Statistical significance of single and interactive effects of sex (S), nitrogen (N), and phosphorus (P) on main parameters based on three-way ANOVA.

Parameter	P > FS	P > FN	P > FP	P > FS × N	P > FS × P	P > FN × P	P > FS × N × P
Root dry mass (g)	***	**	ns	*	**	***	**
Stem dry mass (g)	ns	**	***	ns	*	***	*
Leaf dry mass (g)	**	ns	***	***	ns	***	***
Above-ground dry mass (g)	*	ns	***	**	ns	***	***
Total dry mass (g)	***	ns	***	**	ns	***	***
Total leaf areas (cm^2^)	**	***	**	ns	ns	ns	ns
L/T ratio	ns	***	**	***	ns	ns	**
S/T ratio	***	**	***	***	**	**	ns
S/A ratio	***	***	ns	***	ns	*	*
R/T ratio	***	**	***	*	***	*	ns
R/L ratio	**	***	***	***	***	*	ns
SLA (cm^2^ g^−1^)	**	***	***	***	***	***	***
Leaf C content (mg)	**	***	***	***	ns	***	***
Leaf N content (mg)	ns	***	***	*	**	ns	**
Leaf P content (mg)	ns	***	***	***	*	ns	***
Leaf C/N ratio	**	ns	ns	***	**	***	ns
Leaf C/P ratio	ns	**	***	ns	*	ns	ns
Leaf N/P ratio	ns	***	***	**	ns	***	ns
Leaf starch content (mg)	***	***	***	***	*	***	***
Leaf sucrose content (mg)	***	**	ns	***	*	***	***
Leaf TSS content (mg)	***	*	ns	**	*	***	***
Leaf NSC content (mg)	***	**	**	**	**	***	***
Leaf tannin concentration (mg g^−1^)	**	ns	*	ns	ns	ns	ns
Leaf flavonoid concentration (mg g^−1^)	***	ns	***	***	***	**	ns
Leaf total phenol concentration (mg g^−1^)	ns	ns	*	**	ns	*	ns
Leaf lignin concentration (mg g^−1^)	*	ns	**	ns	*	ns	ns
Leaf cellulose concentration (mg g^−1^)	ns	**	ns	ns	*	ns	ns

FS, the sex effect; FN, the nitrogen effect; FP, the phosphorus effect; FS × N, the interactive effect of sex and nitrogen; FS × P, the interactive effect of sex and phosphorus; FN × P, the interactive effect of nitrogen and phosphorus; and FS × N × P, the interactive effect of sex, nitrogen, and phosphorus. ns, not significant; * *p* < 0.05; ** 0.01 < *p* < 0.001; and *** *p* < 0.001.

**Table 2:** Pearson correlation coefficients between leaf defensive parameters and nutrient and NSC contents of the female (upper triangle) and male (lower triangle, italic) *P. cathayana* seedlings, as affected by different N and P supply.

	CC	PC	NC	C/N	C/P	N/P	TC	FC	LC	CeC	TPC	LDW	StC	SSC	SuC	NSCC
CC		0.932 ***	0.774 **	0.867 ***	−0.346	−0.871 ***	−0.296	−0.028	−0.614 *	−0.332	0.136	0.870 ***	0.653 *	0.344	0.154	0.525
PC	0.912 ***		0.858 ***	0.706 *	−0.642 *	−0.925 ***	−0.306	−0.058	−0.365	−0.434	0.170	0.761 **	0.524	0.218	−0.026	0.376
NC	0.880 ***	0.983 ***		0.360	−0.629 *	−0.703 *	−0.097	0.113	−0.276	−0.686 *	0.526	0.415	0.172	0.026	−0.276	0.087
C/N	0.345	−0.330	−0.136		−0.046	−0.757 **	−0.350	−0.165	−0.677 *	0.021	−0.222	0.951 ***	0.826 **	0.469	0.407	0.691 *
C/P	−0.662 *	−0.853 **	−0.826 **	0.230		0.660 **	0.208	0.214	−0.325	0.583 *	−0.137	−0.115	0.042	0.302	0.561	0.254
N/P	−0.823 **	−0.724 **	−0.633 *	−0.508	0.709 *		0.326	0.163	0.328	0.429	0.022	−0.733 **	−0.531	−0.148	0.065	−0.324
TC	−0.468	−0.590 *	−0.568	0.136	0.569	0.367		0.358	−0.082	0.268	−0.024	−0.344	−0.444	0.136	0.113	−0.066
FC	−0.469	−0.673 *	−0.672 *	0.285	0.809 **	0.567	0.611 *		0.018	−0.138	0.416	−0.224	−0.415	0.395	0.422	0.149
LC	−0.352	−0.452	−0.460	0.133	0.402	0.320	0.551	0.769 **		−0.130	0.025	−0.686 *	−0.546	−0.537	−0.487	−0.635 *
CeC	−0.139	−0.180	−0.210	0.179	0.169	−0.092	0.099	−0.330	−0.558		0.622 *	0.111	0.276	0.271	0.558	0.320
TPC	−0.485	−0.693 *	−0.663 *	0.279	0.682 *	0.424	0.509	0.608 *	0.511	−0.046		−0.207	−0.240	0.114	−0.116	−0.004
LDW	0.946 ***	0.935 ***	0.944 ***	0.096	−0.754 **	−0.732 **	−0.452	−0.584 *	−0.446	−0.140	−0.529		0.878 ***	0.507	0.407	0.741 **
StC	−0.072	0.002	0.001	−0.140	−0.262	−0.178	−0.131	−0.549	−0.654 *	0.637 *	−0.210	0.103		0.373	0.319	0.683 *
SSC	−0.859 ***	−0.960 ***	−0.955 ***	0.080	0.846 **	0.715 **	0.459	0.713 **	0.474	0.072	0.735 **	−0.909 ***	−0.070		0.803 **	0.932 ***
SuC	−0.676 *	−0.878 ***	−0.888 ***	0.303	0.910 ***	0.619 *	0.502	0.887 ***	0.626 *	−0.042	0.724 **	−0.805 **	−0.329	0.902 ***		0.756 **
NSCC	−0.869 ***	−0.958 ***	−0.954 ***	0.058	0.806 **	0.688 *	0.438	0.629 *	0.374	0.169	0.703 *	−0.893 ***	0.082	0.998 ***	0.851 ***	

The variable acronyms are as follows: CC, leaf carbon content; PC, leaf phosphorus content; NC, leaf nitrogen content; C/N, CC/NC ratio; C/P, CC/PC ratio; N/P, NC/PC ratio; TC, leaf tannin concentration; FC, leaf flavonoid concentration; LC, leaf lignin concentration; CeC, leaf cellulose concentration; TPC, leaf total phenol concentration; LDW, leaf dry weight; StC, leaf starch content; SSC, leaf total soluble sugar content; SuC, leaf sucrose content; NSCC, leaf non-structural carbohydrate content. * *p* < 0.05; ** 0.01 < *p* < 0.001; and *** *p* < 0.001.

**Table 3:** Nutrient solution composition of different nitrogen and phosphorus combinations.

Combination	LNLP	LNHP	HNLP	HNHP
KNO_3_	0.4	0.4	5	5
KH_2_(PO_4_)	0.002	1	0.002	1
CaCl_2_	0.07	0.07	0.07	0.07
MgSO_4_·7H_2_O	0.45	0.45	0.45	0.45
H_3_BO_3_	0.0042	0.0042	0.0042	0.0042
MnSO_4_	0.0012	0.0012	0.0012	0.0012
ZnSO_4_·7H_2_O	0.0008	0.0008	0.0008	0.0008
CuSO_4_·5H_2_O	0.00003	0.00003	0.00003	0.00003
Na_2_MoO_4_	0.00004	0.00004	0.00004	0.00004
CoCl_2_	0.00001	0.00001	0.00001	0.00001
FeSO_4_·7H_2_O	0.008	0.008	0.008	0.008
Na_2_EDTA	0.008	0.008	0.008	0.008

Concentrations are in millimolar [48].

## Data Availability

The original contributions presented in the study are included in the article. Further inquiries can be directed to the corresponding author.

## Abbreviations

The following abbreviations are used in this manuscript:

CC	Leaf carbon content
PC	Leaf phosphorus content
NC	Leaf nitrogen content
C/N	CC/NC ratio
C/P	CC/PC ratio
N/P	NC/PC ratio
TC	Leaf tannin concentration
FC	Leaf flavonoid concentration
LC	Leaf lignin concentration
CeC	Leaf cellulose concentration
TPC	Leaf total phenol concentration
LDW	Leaf dry weight
StC	Leaf starch content
SSC	Leaf total soluble sugar content
SuC	Leaf sucrose content
NSCC	Leaf non-structural carbohydrate content
